# Biosensing Platforms for Cardiac Biomarker Detection

**DOI:** 10.1021/acsomega.3c06571

**Published:** 2024-02-20

**Authors:** Zeynep Gerdan, Yeşeren Saylan, Adil Denizli

**Affiliations:** † Department of Biomedical Engineering, Istanbul Beykent University, Istanbul 34398, Turkey; ‡ Department of Chemistry, Hacettepe University, Ankara 06800, Turkey

## Abstract

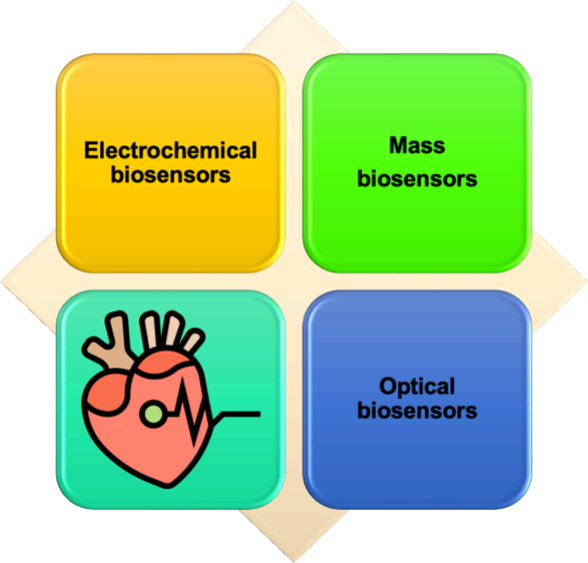

Myocardial infarction (MI) is a cardiovascular disease that occurs when there is an elevated demand for myocardial oxygen as a result of the rupture or erosion of atherosclerotic plaques. Globally, the mortality rates associated with MI are steadily on the rise. Traditional diagnostic biomarkers employed in clinical settings for MI diagnosis have various drawbacks, prompting researchers to investigate fast, precise, and highly sensitive biosensor platforms and technologies. Biosensors are analytical devices that combine biological elements with physicochemical transducers to detect and quantify specific compounds or analytes. These devices play a crucial role in various fields including healthcare, environmental monitoring, food safety, and biotechnology. Biosensors developed for the detection of cardiac biomarkers are typically electrochemical, mass, and optical biosensors. Nanomaterials have emerged as revolutionary components in the field of biosensing, offering unique properties that significantly enhance the sensitivity and specificity of the detection systems. This review provides a comprehensive overview of the advancements and applications of nanomaterial-based biosensing systems. Beginning with an exploration of the fundamental principles governing nanomaterials, we delve into their diverse properties, including but not limited to electrical, optical, magnetic, and thermal characteristics. The integration of these nanomaterials as transducers in biosensors has paved the way for unprecedented developments in analytical techniques. Moreover, the principles and types of biosensors and their applications in cardiovascular disease diagnosis are explained in detail. The current biosensors for cardiac biomarker detection are also discussed, with an elaboration of the pros and cons of existing platforms and concluding with future perspectives.

## 1 Introduction

Cardiovascular diseases, classified as noncommunicable illnesses, are a leading cause of death not only in developed nations but also in underdeveloped countries.^1,2^ According to the World Health Organization, in 2019, there were 17.9 million reported deaths due to cardiovascular diseases, accounting for 32% of all global fatalities. Among these, heart attacks and strokes make up 85% of the fatalities. Projections suggest that by 2030, approximately 23.6 million individuals will succumb to cardiovascular diseases, primarily without experiencing heart attacks and strokes.^3^ Myocardial infarction (MI), a cardiovascular condition, is a major cause of both mortality and morbidity worldwide. Acute MI (AMI) can result in a decreased or interrupted blood supply due to the complete blockage of the coronary artery, often stemming from plaque rupture.^4−7^ This impedes the exchange of nutrients and oxygen between the heart and blood vessels, leading to myocardial cell death and necrosis.^8−10^ AMI is typically diagnosed based on a set of criteria, including (i) characteristic chest pain, (ii) changes in the electrocardiogram (ECG), and (iii) elevated cardiac biomarkers in the bloodstream. The presence of at least two of these three symptoms is sufficient for the definition and diagnosis of AMI.^11,12^ It is crucial to swiftly diagnose patients who have heart attacks upon their hospital admission to ensure prompt treatment. To prevent irreversible damage to cardiac tissues, intervention should be initiated within the first 60 min following the onset of symptoms. For this reason, thrombolytic therapy should commence within 30 min to minimize myocardial necrosis.^13^

The rapid and reliable diagnosis of myocardial infarction (MI) is of paramount importance in clinical settings due to its high mortality and morbidity rates. It is worth noting that a significant proportion of AMI patients may not exhibit changes in their ECG or chest pain. In such cases, the measurement of cardiac biomarkers in the bloodstream plays a pivotal role in the clinical diagnosis of AMI.^14^ These biomarkers, including proteins and various molecules released from damaged myocardial cells, are elevated in the blood circulation, aiding in the early diagnosis of the disease.^15−17^ Given that each cardiac biomarker possesses distinct characteristics such as clinical sensitivity, specificity, release time after symptom onset, clinical cutoff levels, and affinity duration, the rapid, accurate, and simultaneous measurement of these markers can significantly reduce the detection time. This is of utmost importance in saving patients’ lives and minimizing healthcare costs.^18^ The swift and precise diagnosis of an AMI is critical for patient survival. In recent years, biosensors have garnered significant attention for their ability to enable the rapid, accurate, reliable, and specific detection of biomarkers associated with AMI.^19^ Biosensors serve as analytical devices designed to detect specific target analytes such as antibodies, nucleic acids, biological molecules from living organisms, or enzymes. They are typically categorized into three primary groups: electrochemical biosensors, mass biosensors, and optical biosensors. Biosensors offer high sensitivity and selectivity, allowing for real-time detection of target molecules.^20,21^

This review focuses on biosensors for the diagnosis of cardiovascular diseases, particularly myocardial infarction (MI). We begin with an introduction to MI, emphasizing the significance of cardiac biomarkers in medical applications. Subsequently, we provide a comprehensive assessment of the performance and key characteristics of the latest biosensors designed for this purpose. In the concluding section of the review, we offer a concise discussion on the future prospects and potential advancements in this field. There are some novelty and contributions of this review in the context of the state of the art, including contextualization of the problem, introduction of biosensors, biosensor types in cardiovascular disease diagnosis, integration of nanomaterials, and future perspectives. In summary, the novelty and contributions of this review lie in its comprehensive exploration of biosensors, the integration of nanomaterials, and the critical assessment of current technologies for cardiac biomarker detection. The future perspectives also indicate a forward-looking aspect, potentially guiding researchers and practitioners in the field.

## 2 Cardiac Biomarkers

Cardiac biomarkers play a crucial role in the diagnosis, risk assessment, and prognosis monitoring of patients with heart attacks.^22^ Notably, the discovery of troponin subunits by Ebashi and Kodama in 1965 and the subsequent elucidation of troponin’s molecular physiology by Greaser and Gergely in 1972 represented a significant milestone in understanding heart muscle contraction.^23^ Cardiac troponin (cTn) is present in both skeletal and cardiac muscle tissues and comprises three subunits: troponin I, troponin T, and troponin C.^24^ Troponin T and I are highly sensitive and specific to myocardial tissue. When myocytes are damaged, various proteins are released into the bloodstream, with cTn being one of these specific biomarkers indicating myocardial necrosis.^25,26^ Circulating cardiac troponins are used to determine the extent of myocardial infarction and are considered the gold standard due to their superior sensitivity and specificity compared to other biomarkers.^27−29^ Troponin T has a molecular weight of 37 kDa and remains elevated in the blood for up to 14 days following the onset of cardiac ischemia. Its concentration rises to around 50 ng/mL within hours of an AMI. On the other hand, Troponin I, an inhibitory protein within the troponin-tropomyosin complex, consists of 209 amino acids with a weight of 23 kDa. Its isoelectric point is 9.87.^30−34^

In healthy individuals, the concentration of troponin I is typically less than 0.4 ng/mL.^35^ However, within 4–6 h of the onset of an AMI, its blood concentration rises to 50 ng/mL, peaks after 12–24 h, and remains elevated in the blood for a span of 10 to 21 days.^36−38^ These characteristics make cardiac troponins invaluable for the diagnosis and monitoring of myocardial infarction. Troponin C is responsible for binding calcium and magnesium, which are both crucial elements for muscle contraction. It exists as a single isoform present in all striated muscles and is, therefore, not of particular interest to cardiology as it lacks specificity for the myocardium.^39^ Achieving a rapid, sensitive, and accurate diagnosis of troponin levels is critical for preventing excessive and irreversible damage to the heart. In healthy individuals, blood troponin levels typically fall within the range of 20–30 pg/mL. Before cardiac troponin became the gold standard, creatine kinase, which has lower specificity compared to troponin, and its isoenzyme, creatine kinase MB, were utilized as cardiac biomarkers.^40−43^

Creatine kinase (CK)-MB was initially identified as a cardiac biomarker in 1979.^44^ CK-MB levels in heart muscle increase by 5 to 20 times in the bloodstream 5–6 h after the onset of AMI symptoms and return to their normal concentration within 32–72 h. This enzyme has a molecular weight of 86 kDa.^45,46^ However, its low specificity for cardiac muscle, as it is also present in skeletal muscle, limits its clinical utility. Nevertheless, serial measurements of CK-MB are commonly employed for monitoring reinfarction. Elevated CK-MB levels in blood serum following AMI symptoms have been found to correlate with imaging and pathology studies, providing insight into the extent of the infarction.^47,48^ Myoglobin is a cytoplasmic oxygen-binding protein comprising 153 amino acids, with a weight of 17.6 kDa, found in smooth, skeletal, and cardiac muscle.^49,50^ Its small size enables it to begin increasing in blood concentration within 2–3 h when myocardial cell damage commences, reaching levels up to 200 ng/mL. Myoglobin peaks in 4–6 h and then decreases to normal levels of 6–85 ng/mL in the blood after 18–24 h.^51−53^ Its main drawback is its abundance in skeletal muscle, leading to a lack of specificity for cardiac muscle. Therefore, it primarily serves as an indicator of heart damage’s extent, as it is released into the circulation alongside CK-MB and troponin.^54,55^

Human serum albumin, a protein abundant in blood, consists of 585 amino acid residues with a weight of 66.5 kDa and is synthesized by the liver. The last amino-terminal of human serum albumin is unstable and can bind to transition metals such as cobalt and copper from this region.^56,57^ When ischemia occurs, various factors, including hypoxia, oxidative stress, acidosis, and membrane disruption, cause a decrease in albumin’s binding affinity. This results in a change in albumin structure, referred to as ischemia-modified albumin.^58^ Biomarkers such as troponin, CK-MB, myoglobin, and ischemia-modified albumin are highly sensitive to cell necrosis, yet diagnosing myocardial ischemia can be challenging. Prolonged ischemia leads to myocardial cell death, making early diagnosis of myocardial ischemia crucial in reducing the damage caused by ischemia.^59^ C-reactive protein (CRP), one of the best-known diagnostic and prognostic biomarkers, was discovered in 1930.^60^ This acute-phase plasma protein belongs to the short pentraxin family group and is primarily produced by the liver.^61−63^ Although it was initially thought to be produced solely by the liver under the control of interleukin-6, recent studies have shown that CRP is also synthesized in the smooth muscle cells of human coronary arteries, particularly in diseased vessels.^64^ CRP is an inflammation biomarker, with its concentration in human serum increasing several hundred-fold in response to infection, tissue damage, or acute injury.^65^ In healthy individuals, CRP is typically present at concentrations of around 0.8–3 mg/L, but this level increases to approximately 3 μg/mL within hours of MI onset. Compared with cardiac troponin, CRP is less specific and less sensitive as a biomarker of cardiac damage. However, the severity and extent of atherosclerosis, a common cause of AMI, correlate with serum CRP levels.^66,67^

## 3 Biosensors

**Figure 1:**
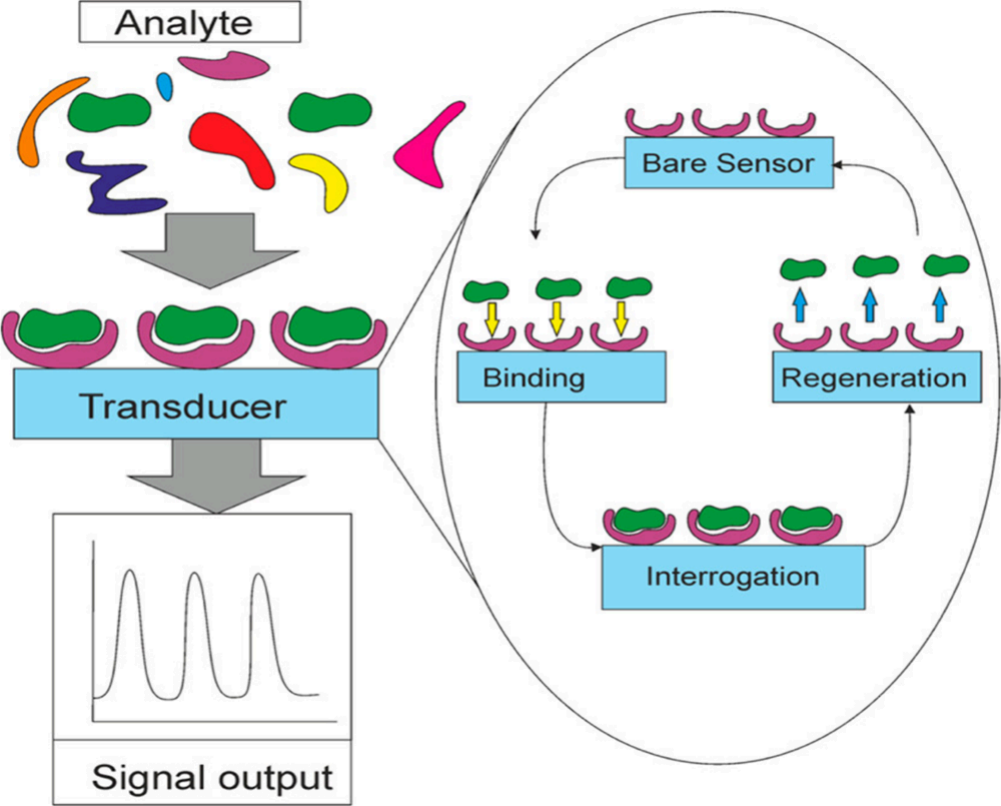
Scheme of biosensor operation and regeneration. Reprinted with permission from ref (74). Copyright 2015 American Chemical Society.

A biosensor serves as a robust analytical tool widely employed for the detection and quantification of target molecules, particularly in medical applications.^68^ In its typical structure, a biosensor comprises three main components: a bioreceptor, a transducer, and an electronic unit.^69^ The transduction principles underlying biosensors can be categorized into various types, including electrochemical, piezoelectric, optical, thermal, micromechanical, magnetic, wireless, and more. The fundamental basis of biosensor operation relies on specific interactions between the analyte and the receptor. This interaction results in a change in one or more properties, such as heat, pH, mass, electron transfer, potential difference, or alterations in optical characteristics, which are subsequently detected by the transducer.^70−73^ As depicted in Figure 1, the biosensor has different steps, including operation and regeneration. After analyte binding and interrogation, regeneration is executed in order to return the biosensor surface and bioreceptor to their original configuration.^74^

Conventional diagnostic methods are often characterized by their time-consuming and expensive nature. Consequently, there is a pressing need for novel detection methods that are fast, reliable, and sensitive. For these reasons, biosensors have gained immense importance as devices for compound detection.^75,76^ In the context of biosensors, the analyte to be detected is identified. The key components responsible for this identification are bioreceptors, also known as recognition elements. Bioreceptors are vital biological elements in biosensors that possess specificity and interact with a single analyte, enabling sensitive and selective analysis. These bioreceptors can take the form of various biological entities, such as enzymes, antibodies, DNA aptamers, whole cells, and more.^77^ Among the most commonly used bioreceptors are enzymes and antibodies.^78,79^ Transducers play a pivotal role in biosensors by converting the sensed form of energy, arising from the interaction between the bioreceptor and the analyte, into another form of energy, typically electrical. This transformation allows for the generation of a measurable signal that correlates with the presence or quantity of a chemical or biological target analyte. Enzymes are frequently employed as biocatalysts to accelerate biological reactions. Enzyme-based biosensors rely on catalytic reactions and coupling abilities to detect target analytes.^80^ Nucleic acids, in the form of aptamers, are short DNA or RNA fragments with unique spatial structures that enable them to bind to specific molecular targets. Aptamers possess advantages such as thermal stability, extended shelf life, and lenient storage and handling requirements due to their chemical structure.^81^ Antibodies are complex proteins composed of two identical light chains and two identical heavy chains. They are employed as bioreceptors in biosensors and their functional outcome is determined by the quality and quantity of antibodies.^82−84^ Antibodies are highly specific in their binding capabilities, making them a favorable choice for inclusion in biosensors.^85^ Living cells express a diverse array of molecules (receptors) at specific ratios, enabling them to not only respond quantitatively to specific stimuli in a given context but also assist in the quantitative analysis of multiple analytes with less effort and expense. Using cells as bioreceptors ensures that the enzymes and other biomolecules required for sensing are present in their native environment, optimizing their activity and specificity toward the target. This unique characteristic allows cells to enhance the functional strategy of biosensors in ways not previously achievable with molecule-based biosensors.^86^ Selectivity is a crucial parameter for biosensors, as they must be able to distinguish analytes in complex matrices of real samples.^87−89^ Electrochemical, optical, and mass transducers are utilized in biosensors to measure and transmit the signals resulting from the interaction between analytes and ligands.^90^ Biosensor platforms find applications across a wide spectrum of fields, including medicine, health technology, pharmacology, and environmental analysis.^91,92^ On the other hand, the introduction of nanomaterials with a size of less than 100 nm into the field of biosensing has led to a rapid increase in the signal amplification and sensitivity of biosensors. Nanomaterials gain new properties by changing their size. Thanks to these new properties of nanomaterials, their chemical, thermal, electrical, optical, and magnetic properties change. It is widely used in optical electrochemical and mass biosensors to amplify signals, increase sensitivity, and obtain a low limit of detection (LOD) value and a large linear field. A superior surface area/volume ratio and a higher catalysis and sensing response provide significant benefits compared to macroscale materials for biological and biomedical applications.^93−97^

### 3.1 Electrochemical Biosensors

Electrochemical biosensors have been a cornerstone in various fields, including metabolism research, control of biological processes, industry, and environmental monitoring. These biosensors operate by converting chemical events into detectable electrical signals using an electrochemical detector during the biointeraction process.^98−100^ Electrochemical biosensors are typically categorized into three main groups based on the measurable parameter, namely amperometric, potentiometric, and impedimetric biosensors.^101,102^ The choice of a specific type of electrochemical biosensor often depends on the application and the specific analyte being detected. These biosensors offer several advantages such as high sensitivity, straightforward sample preparation, rapid response times, affordability, and the potential for miniaturization. However, they are not without their limitations, including narrow linear analyte ranges, inadequate detection limits, or limited selectivity in certain cases.^103−105^ Despite these drawbacks, electrochemical biosensors continue to be valuable tools in a wide range of applications due to their ability to provide real-time and accurate information on various analytes. Amperometric biosensors operate by continuously measuring the current generated during oxidation and reduction reactions in a given process. Clark oxygen electrodes are often used as the basis for amperometric biosensors.^106^ These biosensors are preferred for mass production due to their sensitivity and practicality compared to potentiometric biosensors. The choice between amperometric, potentiometric, or impedance biosensors depends on factors such as the specific analyte of interest, sensitivity requirements, cost, and ease of use. In recent years, there has been a growing interest in impedance biosensors due to their label-free, real-time, and nondestructive nature, particularly in the fields of cell biology and molecular diagnostics.^107^ However, the popularity of these biosensors may change with the emergence of new technologies and applications. Amperometric biosensors have diverse applications and include examples like glucose, lactate, cholesterol, and enzyme-based biosensors.^108^ Potentiometric biosensors, on the other hand, measure the potential difference between the working electrode and reference electrode when the current is close to zero, revealing information about ion activity in the electrochemical reaction.^109^ Some examples of potentiometric biosensors include pH sensors, ion-selective electrodes, and gas sensors. Impedimetric biosensors, the newest among electrochemical biosensors, enable label-free detection and rapid analysis of a wide range of analytes from small molecules to cells. Conductometric biosensors, a subset of impedimetric biosensors, measure the ability to conduct an electrical current between electrodes in a solution and/or reference electrodes. They are often used to investigate enzymatic reactions that lead to changes in ion concentration.^110^ The amperometric biosensors are commonly used due to their simplicity and low LOD. The popularity of these biosensors can vary significantly across different fields and industries.

**Figure 2:**
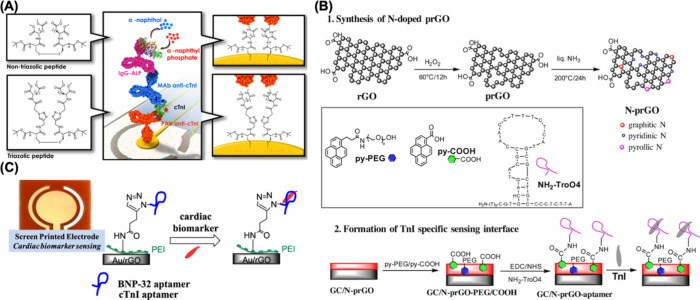
(A) Surface preparation of a gold electrode for cardiac troponin I detection. Reprinted with permission from ref (111). Copyright 2018 Elsevier. (B) Synthesis of nitrogen-doped reduced graphene oxide and its use for cardiac troponin I detection. Reprinted with permission from ref (112). Copyright 2018 Elsevier. (C) Modification of the screen-printed electrodes for cardiac troponin I detection. Reprinted with permission from ref (113). Copyright 2018 American Chemical Society.

As an illustrative example, Dhawan et al. conducted a study on the detection of cardiac troponin I in serum samples. They prepared a biosensor by assembling nontriazole peptides on a gold electrode (Figure 2A). This modification with nontriazole peptides endowed the biosensor with antifouling properties, enabling the successful detection of cardiac troponin I in serum samples at concentrations as low as 1.9 pg/mL. This research demonstrates the potential of biosensors in highly sensitive and specific analyte detection, particularly in the context of critical biomarkers like cardiac troponin I.^111^ In a related study, Chekin et al. developed an electrochemical biosensor designed for the detection of troponin I. The study showcased the significant promise of electrodes modified with nitrogen-doped reduced graphene oxide when applied to serum and saliva samples. This biosensor exhibited a remarkable LOD of 1 pg/mL, highlighting its suitability for routine clinical monitoring of troponin I, as depicted in Figure 2B.^112^ This research underscores the potential of advanced materials and biosensor technologies in the field of medical diagnostics. In a study by Grabowska et al., they designed an aptamer-based electrochemical biosensor for the detection of troponin I (Figure 2C). Their approach aimed to create a multianalyte detection platform for various cardiac biomarkers. In this assay, gold-based screen-printed electrodes were modified with graphene oxide. For the detection of troponin I, they achieved a linear response range from 1 pg/mL to 10 ng/mL, with an impressively low LOD of 0.001 pg/mL.^113^ This research showcases the potential of aptamer-based biosensors for highly sensitive and specific detection of cardiac biomarkers such as troponin I, which is vital in the diagnosis and monitoring of cardiac conditions.

Centi et al. developed a disposable electrochemical biosensor for the detection of C-reactive protein (CRP) in serum samples. This biosensor was created by using a combination of magnetic particles and carbon-based screen-printed electrodes. The surface of the biosensor was modified by adding the RNA aptamer to a monoclonal antibody and incorporating alkaline phosphatase. All measurements were conducted using differential pulse voltammetry, and the biosensor achieved an LOD of 0.054 mg/L for CRP in serum samples.^114^ This research presents a valuable tool for the detection of CRP, which is a crucial biomarker for inflammation and a wide range of medical conditions. O’Regan et al. developed an amperometric biosensor for the rapid detection of myoglobin. This biosensor utilizes a one-step indirect sandwich analysis approach. The study demonstrated that the test is accurate, with a coefficient of variation of less than 8%.^115^ Such biosensors offer a valuable means of quickly and accurately assessing the presence of myoglobin, which is a significant biomarker associated with various medical conditions, particularly those related to muscle and heart health. Sun et al. developed biosensors using gold nanoparticles for the detection of myoglobin. The resulting electrode was characterized by electrochemical impedance spectroscopy and cyclic voltammetry. They evaluated the response of the gold-myoglobin surface in a buffer solution and determined an LOD of 2.7 ng/mL for myoglobin in the concentration range of 10 ng/mL to 1 μg/mL.^116^ These biosensors demonstrate the potential for sensitive myoglobin detection, which has clinical significance in various medical contexts, particularly in assessing muscle and heart health. Zhang et al. presented an electrochemical biosensor for the detection of myoglobin. This biosensor was constructed on a carbon-fiber microelectrode modified with antibodies. The study reported an impressive LOD of 1.2 pg/mL within a linear range of 0.005–20 ng/mL under optimal conditions.^117^ Such highly sensitive biosensors have the potential to play a critical role in the early detection of myoglobin, which is a vital biomarker associated with muscle and heart health. Table 1 depicts the comparison of these electrochemical biosensors for different cardiac biomarker detection.

**Table 1:** Comparison of Electrochemical Biosensors for Cardiac Biomarker Detection

Biomarker	Modification	Time	Reusability	LOD	Range	Real sample	ref
Troponin I	Peptide dendrons on gold electrodes	30 min	Not available (NA)	1.9 pg/mL	10–100 pg/mL	Serum	(111)
Troponin I	Nitrogen-doped porous reduced graphene oxide	NA	10 times	1 pg/mL	1 pg/mL to 10 ng/mL	Serum and saliva	(112)
Troponin I	Polyethylenimine/reduced graphene oxide films	NA	10 times	0.001 pg/mL	1 pg/mL to 10 ng/mL	Serum	(113)
C reactive protein	Magnetic particles and carbon-based screen-printed electrodes	5 min	NA	0.054 mg/L	0.1–50 mg/L	Serum	(114)
Myoglobin	Antihuman cardiac myoglobin antibody	5–60 min	2 times	NA	85–925 ng/mL	Whole blood	(115)
Myoglobin	Gold nanoparticles	15 min	NA	2.7 ng/mL	10 ng/mL to 1 μg/mL	Synthetic serum	(116)
Myoglobin	Antibody-modified carbon fiber microelectrode	35 min	5 times	1.2 pg/mL	0.005–20 ng/mL	Human serum	(117)

### 3.2 Mass Biosensors

The quartz crystal microbalance (QCM) is a mass-based piezoelectric biosensor that finds extensive use in label-free biosensing applications.^118^ QCM biosensors operate by measuring minute changes in mass adsorbed on the surface of a quartz crystal disc, resulting in a shift in resonance frequency.^119,120^ The performance of QCM biosensors, including their high sensitivity, low energy consumption, and ease of replacement, is heavily dependent on the physical properties and chemical structure of the material coated on the active electrode. The mass and viscoelastic properties of this coated material influence the response of the QCM biosensor. In recent years, QCM biosensors have garnered significant attention due to their cost-effectiveness and the ability to deposit a wide range of materials.^121,122^ Despite their advantages, QCM biosensors have some limitations, including baseline instability in response to fluctuations in ambient humidity and temperature and changing environmental conditions. They may also involve complex circuitry, leading to potential interference between channels and requiring more maintenance and calibration.^123^ These biosensors have been applied across a diverse array of implementations for the detection of various targets, including hormones, bacteria, cells, viruses, and more.^124−127^ Their versatility and ability to provide real-time, label-free detection make them valuable tools in the fields of diagnostics, environmental monitoring, and research.

**Figure 3:**
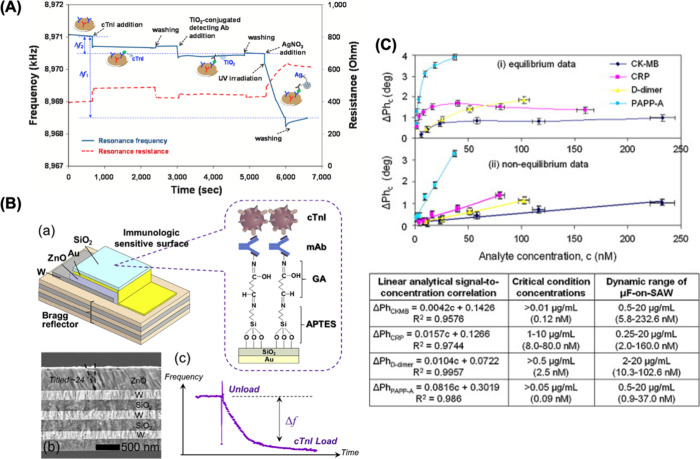
(A) Response of QCM biosensor with adding cTnI in human serum captured by antibody-TiO2 conjugates and the subsequent photocatalytic silver staining process. Reprinted with permission from ref (130). Copyright 2021 Elsevier. (B) Scheme of cardiac biomarker detection (a), SEM image of ZnO piezoelectric film (b), and sensing mechanism of biosensor (c). Reprinted with permission from ref (131). Copyright 2020 Elsevier. (C) Curves of four cardiac biomarkers as extracted from (i) equilibrium analysis and (ii) pre-equilibrium phase responses for CK-MB and CRP, and 600 s for D-dimer and PAPP-A. Reprinted with permission from ref (132). Copyright 2011 Elsevier.

Agafonova et al. developed a QCM biosensor for the detection of myoglobin. This biosensor was designed to detect the interaction between cardiac myoglobin and monoclonal antibodies, and the detection could be directly monitored. They immobilized monoclonal antibodies, which specifically reacted against a single antigenic determinant by drop-casting them onto a gold surface. The biosensor they designed enables analysis on the electrode’s surface without the need for additional chemical modifications or multiple labeling. The analysis is conducted in real-time and is a one-step online process. Importantly, this biosensor allows for the examination of binding from plasma samples in as little as 30 to 120 s.^128^ This research showcases the potential of QCM biosensors for the rapid and label-free detection of myoglobin, which is a critical biomarker for assessing cardiac health. Wong-ek et al. enhanced a QCM biosensor for the detection of troponin T. In their approach, they immobilized antibodies by functionalizing the biosensor with a polyvinyl chloride doped surface using the spray coating technique. This modification improved the sensitivity of the QCM biosensor compared with an uncoated surface. They observed that the frequency shift was directly proportional to the troponin T concentration, and the biosensor achieved an LOD of 5 ng/mL.^129^ This study demonstrates the potential of QCM biosensors in the sensitive detection of troponin T, a key cardiac biomarker used in diagnosing heart-related conditions. Lim et al. introduced a highly sensitive and reproducible QCM biosensor for the detection of troponin I in a concentration range of 25 pg/mL to 15 ng/mL. To enhance the sensitivity of the biosensor, they employed signal amplification techniques involving the use of titanium oxide nanoparticles with photocatalytic silver staining.^130^ The frequency decrease represents an increase in the effective mass on the surface of the QCM biosensor. The frequency change (Δfrequency) is indicated by the double-ended arrow in Figure 3A. Δf1 and Δf2 denote changes in the resonance frequencies in the TiO_2_ nanoparticle-conjugated sandwich immunoassay without and with signal amplification by the photocatalytic silver staining reaction, respectively. This research highlights the potential of QCM biosensors in detecting troponin I in a highly sensitive and specific manner, which is of great significance in cardiac diagnostics and monitoring. Liu et al. developed a microfabricated thickness shear-mode electroacoustic biosensor based on a zinc oxide (ZnO) piezoelectric film for the detection of cardiac troponin (as depicted in Figure 3B). This biosensor exhibits an impressive LOD of 20 pg/mL for troponin I and provides results in less than 2 min. The biosensor demonstrates high specificity and sensitivity when tested with rabbit and clinical serum samples.^131^ This innovative biosensor offers a rapid and highly sensitive means of detecting cardiac troponin, which is a critical biomarker for diagnosing heart-related conditions. Mitsakakis et al. presented an innovative approach by integrating a multichannel microfluidic module with a surface acoustic wave (SAW) biosensor for the simultaneous detection of four cardiac biomarkers: creatine kinase myoglobin (CK-MB), C-reactive protein (CRP), D-dimer, and pregnancy-associated plasma protein A (PAPP-A). This biosensor comprises two main components: the SAW part and the microfluidic module. As demonstrated in Figure 3C, the biosensor achieved an impressive LOD with concentrations below 0.12 μg/mL within the range of 0.25–20 μg/mL for these biomarkers which corresponding to the subsequent values in molar (M) units as 5.8–232.6 nM for CK-MB, 2.0–160.0 nM for CRP, 10.3–102.6 nM for D-dimer, and 0.9–37.0 nM for PAPP-A.^132^ This integration of SAW technology and microfluidics provides a promising platform for the efficient and sensitive detection of multiple cardiac biomarkers, which is essential in clinical diagnostics. Table 2 shows the comparison of these mass biosensors for different cardiac biomarker detection.

**Table 2:** Comparison of Mass Biosensors for Cardiac Biomarker Detection

Biomarker	Modification	Time	Reusability	LOD	Range	Real sample	ref
Myoglobin	Monoclonal antibodies	30–120 s	NA	34.2 ng/mL	34.2–394.2 ng/mL	Plasma serum	(128)
Troponin T	Polyvinyl chloride-doped COOH	60 min	NA	5 ng/mL	5–50000 ng/mL	NA	(129)
Troponin I	Titanium oxide nanoparticle	6000 s	3 times	18 pg/mL	25 pg/mL to 15 ng/mL	Human serum	(130)
Troponin I	ZnO film	<2 min	NA	20 pg/mL	0.04–2 ng/mL	Rabbit and human serum	(131)
C reactive protein	Polydimethylsiloxane- based microfluidic module	<30 min	NA	0.12 μg/mL	0.25–20 μg/mL	Blood plasma	(132)

### 3.3 Optical Biosensors

Optical biosensors have gained significant attention in recent years due to their ability to provide real-time detection, easy naked-eye recognition, and the advantage of requiring less complex equipment for analysis.^133^ These biosensors offer several advantages over conventional methods, including low cost, rapid analysis times, and suitability for on-site analysis.^134^ They are compact devices that operate by utilizing a receptor and an optical converter.^135^ The primary function of optical biosensors is to generate optical signals that are proportionate to surface optical properties resulting from the interaction between the analyte and the recognition element in the sensing environment.^136^ Optical biosensors can utilize a variety of biorecognition elements, including antibodies, proteins, and nucleic acids.^137−139^ These biosensors encompass a wide range of analytical methods based on the interaction of light with matter and often allow for continuous and simultaneous monitoring of multiple analytes.^140^ However, it is worth noting that optical biosensors can be more expensive to manufacture and operate compared to electrochemical biosensors. They may also have limitations in terms of compatibility and reproducibility.^141^ The growing interest in optical biosensors is driven by the competitive advantage they offer in terms of sensitivity when compared to other detection methods.^142^

Surface plasmon resonance (SPR) biosensors, as one of the most preferred optical biosensors, have been the focal point of research for the past 25 years, especially in the study of biomolecular interactions.^143^ Commercialized SPR biosensor technology finds a wide range of applications in fields such as determining affinity and binding constants, genotype analysis, environmental and food analysis, medical diagnostics, and biomedical research.^144−147^ These biosensors are extensively used in the analysis of various substances, including antibodies, proteins, lipids, viruses, and in drug discovery.^148−152^ One of the key advantages of SPR biosensors is their ability to perform label-free, rapid, and selective detection without the need for preliminary purification steps.^153^ However, they do have certain challenges that can affect their use in specific applications. These challenges include complexity, cost, and the requirement for skilled operators for setup, calibration, and maintenance. The initial acquisition cost for an SPR biosensor can be relatively high, which may present a barrier for smaller laboratories or researchers. Additionally, SPR instruments are typically bulkier and less portable than some other optical biosensors. Certain sample types, such as highly turbid or viscous liquids, may not be suitable for SPR measurements. Moreover, samples that strongly absorb in the visible or near-infrared range can interfere with the SPR signals. Despite these limitations, SPR biosensors continue to be highly valuable in various research and diagnostic applications. Researchers and developers should consider these disadvantages when selecting the most appropriate biosensor technology for their specific needs and design experiments to address potential challenges.^154,155^

**Figure 4:**
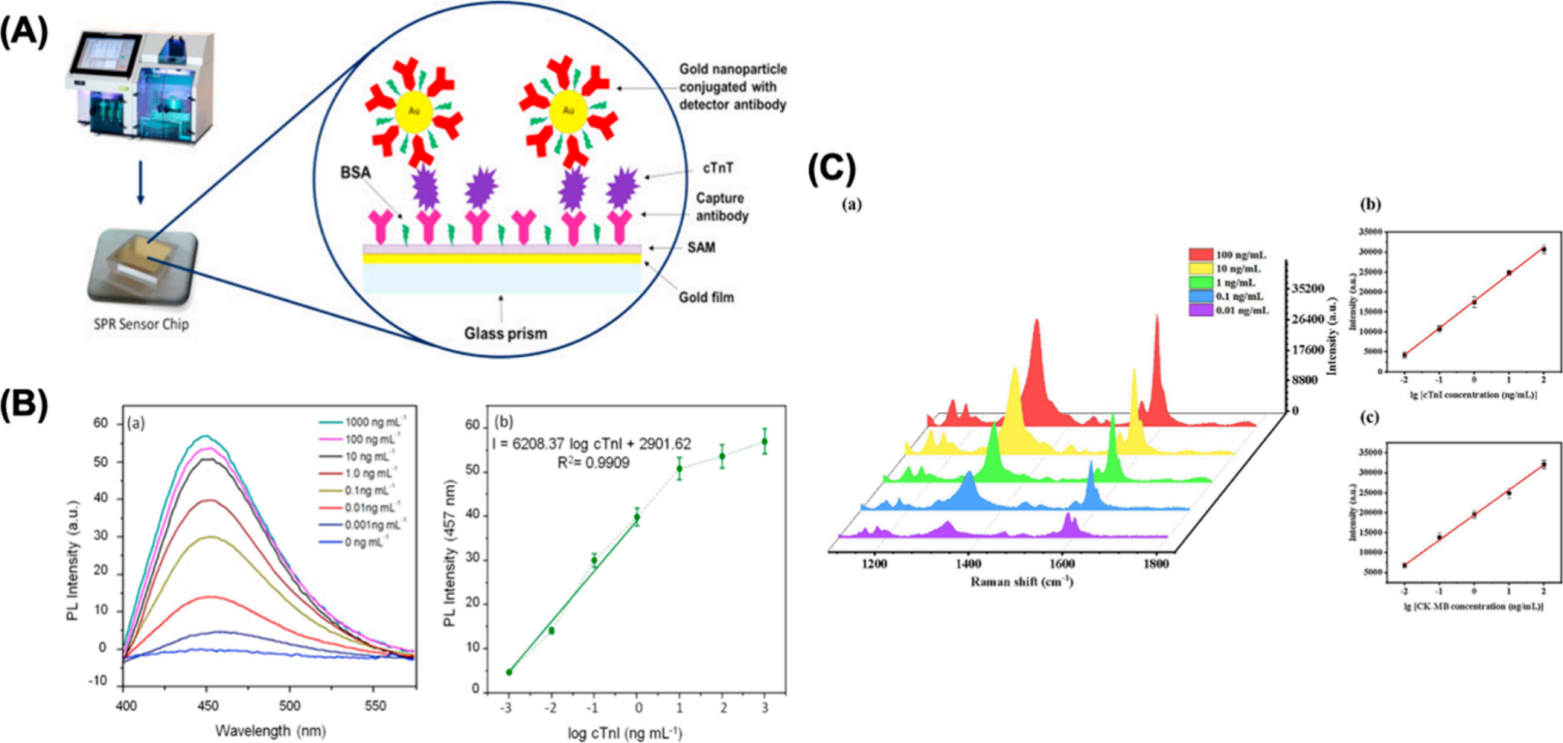
(A) Scheme of SPR sensor utilizing gold nanoparticles conjugated with a detector antibody. Reprinted with permission from ref (157). Copyright 2016 Elsevier. (B) The fluorescence spectra of the biosensor with increasing concentrations of cTnI antigen (a). The relationship between the PL intensity of the biosensor with increasing log concentrations of cTnI (b). Reprinted with permission from ref (166). Copyright 2016 Elsevier. (C) SERS spectra corresponding to the different concentrations of cTnI and CK-MB (a). The relation curves of the Raman intensity versus the logarithm of the concentration of cTnI and CK-MB (b and c). Reprinted with permission from ref (173). Copyright 2021 Elsevier.

Dutra et al. utilized an SPR biosensor for the real-time and rapid detection of human troponin T. They improved the SPR biosensor by using a streptavidin-terminated self-assembled monolayer to immobilize biotinylated antitroponin T monoclonal antibodies. The result of this assay demonstrated that the improved SPR biosensor had a detection range for troponin T of 0.03 to 6.5 ng/mL. This enhanced SPR biosensor exhibited a rapid response time of as little as 800 s and allowed for the specific and reproducible detection of troponin T in human serum.^156^ This research underscores the utility of SPR biosensors in the sensitive and specific detection of cardiac biomarkers, such as troponin T, which is crucial in the diagnosis of heart-related conditions. Pawula et al. developed an SPR biosensor for the fast and selective detection of troponin T, as shown in Figure 4A. They optimized this SPR biosensor using both direct and sandwich immunoassay methods. The process involved surface modification with self-assembled monolayers (SAM) on a clean biosensor surface. Various concentrations of 11-mercaptoundecanoic acid (MUDA) were investigated and added to the biosensor surface until the solution was completely covered. Antibody immobilization was carried out after the appropriate pH for the anti-cTnT 1C11 antibody. Subsequently, the surface was cocoated with a capture antibody and SAM, and various binding techniques were explored. Prebinding testing was conducted before conducting direct and sandwich assays. To amplify the signal obtained through a sandwich assay, gold nanoparticles were introduced and conjugated with anti-cTnT 7G7 detector antibodies. The actual binding responses for cTnT detection antibodies were obtained after subtracting the nonspecific binding responses obtained for the control antibody. This signal enhancement allowed for the detection of cTnT concentrations as low as 0.5 ng/mL in 50% human serum.^157^ This research highlights the potential of SPR biosensors for highly sensitive and selective detection of cardiac biomarkers, such as troponin T, in clinical samples. Çimen et al. developed an SPR biosensor for the detection of troponin I. The biosensor’s surface was immobilized with anticardiac troponin I monoclonal antibody. The characterization of the prepared biosensor was conducted by using ellipsometry, atomic force microscopy, and contact angle analysis. Selectivity tests were carried out through the competitive adsorption of myoglobin, immunoglobulin G, and prostate-specific antigen. The biosensor exhibited an LOD of 0.00012 ng/mL, and its reproducibility was tested over five different days and within the same day, highlighting its precision and sensitivity for troponin I detection.^158^ This study demonstrates the potential of SPR biosensors for the highly sensitive and specific detection of cardiac biomarkers such as troponin I, which is crucial for diagnosing heart-related conditions. Atay et al. created an SPR biosensor for myoglobin detection using a molecularly imprinted polymer (MIP) as the recognition element. They characterized the prepared SPR biosensors and conducted kinetic analyses. The biosensor exhibited an LOD of 4.72 ng/mL, making it suitable for the detection of myoglobin in relevant samples.^159^ This research underscores the potential of MIP-based SPR biosensors for detecting specific biomolecules, such as myoglobin, with reasonable sensitivity. Wolf et al. developed a combined assay for the detection of CRP using macromosaic immunoassays and self-regulating microfluidic networks. This innovative approach allowed the quantitative detection of CRP at a concentration of 30 ng/mL within a rapid time frame of 10 min.^160^ This research demonstrates the potential for the sensitive and fast detection of CRP, which is a crucial biomarker for inflammation and various medical conditions. Choudhary et al. developed a point-of-care SPR biosensor for the sensitive and real-time detection of cTnI using epitope-imprinted molecular receptors. The biosensor’s surface was characterized using fluorescence microscopy and dynamic light scattering techniques. Various techniques, including atomic force microscopy, electrochemical impedance spectroscopy, square wave voltammetry, and cyclic voltammetry, were employed in the fabrication of the SPR biosensor. This portable SPR biosensor demonstrated a low LOD of 0.52 ng/mL and a detection range spanning from 0.78 to 50 ng/mL.^161^ This research showcases the potential of point-of-care SPR biosensors for the rapid and sensitive detection of cardiac biomarkers, specifically cTnI, which is vital in diagnosing heart-related conditions.

Fluorescence is indeed the emission of light from a substance when it absorbs light of the appropriate wavelength. It plays a crucial role in many biosensors, particularly those based on protein interactions. Fluorescent proteins and various mechanisms, such as fluorescence switching and fluorescence resonance energy transfer (FRET), have expanded the capabilities of biosensors, enabling the detection of a wide range of biomolecules with high sensitivity and selectivity. These advancements have revolutionized fields such as molecular biology, medical diagnostics, and environmental monitoring, making fluorescence-based biosensors a valuable tool for researchers and practitioners.^162,163^ Chen et al. developed a sensitive fluorescence biosensor for the detection of myoglobin. They used carbon dots and deoxyribonuclease I-assisted target recycling fluorescence response intensification in this biosensor. In this study, myoglobin aptamer was bound to carbon dots, and the resulting carbon-dot aptamer compound caused quenching of fluorescence emission intensity. The addition of a myoglobin molecule restored the fluorescence intensity. Thus, myoglobin was detected in human urine, saliva and serum diluted with buffer solution over a wide range from 50 pg/mL to 100 ng/mL with a detection limit of 20 pg/mL.^164^ Liu et al. designed an optical biosensor for rapid and accurate detection of AMI. In this study, they developed a fluorescence biosensor based on a graphene oxide platform for cardiac troponin I detection. Fluorescent antitroponin I was bound to graphene oxide, and fluorescence was quenched by this binding. The fluorescence of the fluorescent anti-cTnI aptamer was then restored by adding troponin I. A low detection limit of 0.07 ng/mL was calculated in the concentration range of 0.1–6 ng/mL in human serum. In selectivity analyses, the biosensor exhibited very high selectivity toward troponin I.^165^ Bhatnagar et al. developed an optical biosensor of amine functionalized graphene quantum dots (afGQDs) conjugated with troponin I (anti-cTnI). The conjugated afGQDs were characterized by zeta potential, UV–vis spectroscopy, and field emission scanning electron microscopy. The sensing performance of the biosensor was studied according to the changes in the photon number and photoluminescence of GQDs upon interaction with the anti-cTnI antibody. As shown in Figure 4B, the biosensor exhibited a linear response to cTnI in blood serum from 0.001 to 1000 ng/mL with a detection limit of 0.192 pg/mL.^166^ Lee et al. designed a lateral flow immunoassay (LFIA) system containing fluorescent dye-doped nanoparticles for the sensitive detection of cardiac troponin I. For LFIA, fusion 5 membrane was used to avoid the need for additional matrix, and then the TR-FRET technique was integrated with fusion 5 membrane-based LFIA strip. Silica nanoparticles were synthesized as raspberry-type particles as a fluorescence donor. Gold nanorods were used as fluorescent acceptor particles. The developed TR-FRET based LFIA system performed a sensitive analysis of troponin I in human serum samples with a detection limit of 97 pg/mL.^167^

Raman spectroscopy is an optical measurement technique that can be utilized to analyze inelastically scattered light from a sample material. The energy from the light particle is transferred to the molecules in the material and the remaining power is emitted in what is called inelastic light scattering.^168^ Raman spectroscopy works based on the inelastically scattered ray generated from the vibrational modes of molecular bonds upon laser excitation.^169^ Raman spectroscopy can deliver high molecular specificity, rapid implementation and low to moderate cost.^170^ El-Said et al. prepared indium tin oxide (Ag NPT/ITO) substrates modified with Ag nanopine tree film using electrochemical deposition of Ag from aqueous silver nitrate solution for the detection of myoglobin. They analyzed the optical properties of the Ag NTP/ITO substrate by UV–vis spectroscopy. The activities of different Ag nanostructures/ITO substrates were investigated by using rhodamine 6G dye. The biosensor prepared for the detection of myoglobin exhibited a low detection limit of 10 × 10^–9^ g/mL in the range of 5 × 10^–6^ to 10 × 10^–9^ g/mL.^171^

SERS methods utilize the significant Raman scattering amplification afforded by the close association of Raman-active dyes or biomarkers with plasmonic metal nanostructures to detect targets of interest sensitively and specifically.^172^ Wang et al. developed a microcavity-based SERS biosensor for CK-MB and cTnI detection. Anti CK-MB and anti-cTnI monoclonal antibodies were immobilized on the microcavity-based SERS biosensor together with gold nanoparticles to form an immune chip on the Au–PS-PDA microcavity substrate. With the addition of the target antigen, they formed a capture chip-target antigen-SERS signal probe. The significantly increased SERS optical signal was able to detect cTnI and CK-MB sensitively. The LOD value of the biosensor for cTnI was calculated as 3.16 pg/mL and 4.27 pg/mL for CK-MB.^173^ Zhang et al. designed a fast and sensitive optical biosensor for the detection of CK-MB, troponin I, and myoglobin. Silver–gold core–shell SERS nanotags characterized in transmission electron microscopy for quantitative lateral flow assay (LFA) were used to prepare a multiplex LFA (SERS LFA) based biosensor. Detection antibodies for the three biomarkers were conjugated with SERS nanotags in order of priority, and three test lines were produced on nitrocellulose (NC) membrane for multiplex sensing. After flow of the sample from the sample pad to the absorption pad, the Raman signals of the three test lines are quantified for the quantification of cardiac biomarkers. Owing to the high surface area/volume ratio of the porous NC membrane and the powerful signal of the SERS nanotags, an ultrasensitive LFA with a wide LDR was performed. LODs for Myo, cTnI and CK-MB were calculated to be below the clinical thresholds of 3.2, 0.44, and 0.55 pg/mL, respectively.^174^ Fu et al. designed an optical biosensor for cardiac troponin I detection by immunoassay, AuNPs conjugated with graphene oxide, and malachite green isothiocyanate, a Raman reporter, to produce SERS nanoarrays, which were further conjugated with rabbit polyclonal cTnI antibodies. Magnetic beads were used to form the sandwich structure, and the magnetic beads used were functionalized with mouse monoclonal cTnI antibodies to assemble the capture probe. The LOD was reported to be 5 pg/mL over a range of cTnI concentrations from 0.01 to 1000 ng/mL.^175^Table 3 shows the comparison of these optical biosensors for different cardiac biomarker detection.

**Table 3:** Comparison of Optical Biosensors for Cardiac Biomarker Detection

Biomarker	Detection technique	Time	LOD	Range	Real sample	Ref
Troponin T	Carboxymethyldextran-modified gold chip	<10 min	0.01 ng/mL	0.03–6.5 ng/mL	Human serum	(156)
Troponin T	Gold nanoparticles	3 min	0.5 ng/mL	0.5–40 ng/mL	Human serum	(157)
Troponin I	Gold surface	13.30 min	0.00012 ng/mL	0.001–8.0 ng/mL	Serum	(158)
Myoglobin	Nanoparticles	NA	4.72 ng/mL	0.3–1.0 μg/mL	Serum	(159)
C reactive protein	Self-regulating microfluidic	NA	30 ng/mL	20–500 μg/mL	Human plasma	(160)
Troponin I	NanoMIP	10–15 min	0.52 ng/mL	0.78–50 ng/mL	NA	(161)
Myoglobin	Carbon dots	NA	20 pg/mL	50 pg/mL to 100 ng/mL	Human urine, saliva, and serum	(164)
Troponin I	Graphene oxide	15 min	0.07 ng/mL	0.1–6 ng/mL	Serum	(165)
Troponin I	Graphene quantum dots	10 min	0.192 pg/mL	0.001–1000 ng/mL	Blood serum	(166)
Troponin I	Raspberry-type europium particle	NA	97 pg/mL	NA	Serum	(167)
Myoglobin	3D silver anisotropic nanopinetree array	NA	10 × 10^–9^ g/mL	5 × 10^–6^ to 10 × 10^–9^ g/mL	Urine	(171)
Troponin I, CK-MB	Gold nanoparticles	NA	3.16 pg/mL, 4.27 pg/mL	NA	NA	(173)
CK-MB, troponin I, myoglobin	Plasmonic nanoparticles	15 min	3.2, 0.44, 0.55 pg/mL	0.01–500 ng/mL 0.01–50 ng/mL 0.02–90 ng/mL	Serum	(174)
Troponin I	Graphene oxide/gold nanoparticle	NA	5 pg/mL	0.01–1000 ng/mL	Serum	(175)

It is important to note that the choice of biosensor type depends on the specific application and the characteristics of the target analyte, and each type of biosensor has its advantages and disadvantages. Researchers and developers select the appropriate biosensor technology based on the requirements of the intended use. Moreover, it is also important to consider the specific requirements of the application when choosing between these biosensor technologies. Table 4 describes basic advantages and disadvantages of electrochemical, mass, and optical biosensors.

**Table 4:** Comparison of the Electrochemical, Mass, And Optical Biosensors

Biosensor type	Advantages	Disadvantages
Electrochemical	High sensitivity, easy sample preparation, fast response, low cost, more compact and portable, ease of miniaturization	Narrow analyte range, insufficient detection limit, insufficient selectivity, high sample requirement
Mass	High sensitivity, wide analyte range, low energy use, easy to replace, rapid, longer lifespan	More sensitive to changes in environmental conditions, complex circuitry, interference between channels, requires more maintenance and calibration
Optical	Real-time detection, multianalyte detection, label-free detection, high sensitivity, short detection time, minimal sample preparation	Low compatibility, low reproducibility, expensive

## 4 Conclusion and Future Perspectives

Biosensors have emerged as a promising platform for cardiac biomarker detection in the diagnosis of cardiovascular diseases, particularly in case of myocardial infarction. Through a critical examination of current research, we discuss the advantages and challenges associated with nanomaterial-based biosensing platforms. Real-world applications in healthcare are highlighted, demonstrating the versatility and broad impact of these innovative systems. Additionally, the review explores the integration of nanomaterials in emerging biosensor technologies including wearable devices and point-of-care diagnostics. Electrochemical, mass, and optical biosensors have been prepared to detect different cardiac biomarkers with high sensitivity and specificity. However, despite the progress made in this field, there are still limitations and challenges that need to be addressed, such as the need for better integration with clinical workflows and the optimization of biosensor performance. Moreover, enhancing sensitivity is crucial, particularly for the detection of low-abundance analytes or trace levels of contaminants. Improving the signal-to-noise ratio and decreasing detection limits are ongoing challenges. Achieving high specificity, especially in complex sample matrices, is a persistent challenge. Reducing the cross-reactivity and interference from similar compounds remains a priority. In addition, simplifying and automating sample preparation processes can streamline biosensor assays and reduce the risk of errors. Integrating sample preparation steps into biosensor platforms is an ongoing challenge. The ability to simultaneously detect multiple analytes in a single assay is highly desirable for many applications. Developing biosensors capable of multiplexing, while maintaining accuracy and sensitivity, is an area of active research. Furthermore, creating compact, portable biosensors suitable for point-of-care and field applications is a continuing goal. Minimizing the size, weight, and power requirements of biosensor devices is a challenge for technology developers. For many biosensors, regeneration of the sensing surface after analyte binding is a critical need. Developing efficient and reproducible regeneration strategies is a challenge to extend the lifespan and reduce operating costs. Biosensor stability over time and under different environmental conditions is essential. Addressing issues related to sensor surface aging and performance degradation is an ongoing concern. Ensuring that biosensors are biocompatible and can be safely used in biological and clinical settings is crucial. Avoiding adverse interactions with living organisms and tissues is a challenge in the development of implantable biosensors. Reducing the cost of biosensor production and operation is essential to making them more accessible to a wider range of users and applications. Effectively processing and interpreting the data generated by biosensors, particularly in real-time and continuous monitoring, presents challenges. Developing user-friendly software and algorithms for data analysis is vital. Thus, although biosensors have revolutionized many fields with their capabilities, addressing these limitations and challenges will be key to their continued success and broader adoption. Ongoing research and innovation in the biosensor field are likely to lead to improved sensitivity, specificity, and usability, making biosensors increasingly valuable tools in healthcare, environmental monitoring, food safety, and many other applications. Many diagnostic scenarios require the simultaneous detection of multiple biomarkers. Developing biosensors capable of multiplexed measurements is essential for comprehensive disease diagnosis and monitoring. Biosensors often work well in controlled laboratory conditions but may face challenges when applied to complex, real-world patient samples. Issues like sample matrix interference and variability in sample composition must be addressed. Despite their potential, biosensors face barriers to widespread commercialization. Reasons include high development costs, the need for regulatory approvals, competition with existing diagnostic methods, and the challenges associated with scaling up production. The cost-effectiveness and accessibility of biosensors, especially in resource-limited settings, remain significant challenges. Reducing costs while maintaining performance is essential for broader adoption. Developing standardized protocols for biosensor calibration and validation is crucial to ensuring accurate and reproducible results across different devices and laboratories. Biosensors must navigate regulatory pathways for approval in clinical and diagnostic settings, which can be time-consuming and costly. Overcoming these challenges is essential for realizing the full potential of biosensor technologies in healthcare, diagnostics, and various other fields. Collaborations among researchers, industry, and regulatory bodies are instrumental in advancing the commercial adoption of these innovative technologies.

The widespread adoption of hand-held devices, particularly smartphones, has revolutionized various aspects of daily life. These devices, known for their portability and versatility, have transformed into multifunctional tools with applications ranging from communication to navigation, health monitoring, and entertainment. Smartphones play a crucial role in democratizing access to information, breaking down barriers to knowledge, and fostering a globally connected community. The integration of advanced technologies like artificial intelligence and augmented reality has further expanded the capabilities of hand-held devices, offering intelligent features and immersive experiences. However, the ubiquity of smartphones also raises concerns, including issues related to privacy, digital addiction, and environmental impact.

The future of biosensors for the diagnosis of cardiovascular diseases looks promising. Researchers are exploring new biomarkers and developing new types of biosensors with enhanced performance and improved capabilities. In the near future, it is expected that the biosensors will become more affordable, user-friendly, and widely available, which will enable their integration into clinical practice. The development of biosensors for the real-time monitoring of cardiac biomarkers will also be a major research direction. Additionally, the integration of biosensors with artificial intelligence and machine learning algorithms will enable the development of personalized diagnosis and treatment strategies. These future perspectives are likely to have a significant impact on the early diagnosis and treatment of cardiovascular diseases, improving patient outcomes, and reducing mortality rates. In conclusion, this review not only consolidates the existing knowledge surrounding nanomaterial-based biosensing but also provides insights into future directions and challenges. The synergistic combination of nanomaterials and biosensing technologies holds immense promise for the development of next-generation sensing platforms, fostering advancements in medical diagnostics, environmental surveillance, and beyond.
